# Description of a new deep-water dogfish shark from Hawaii, with comments on the *Squalusmitsukurii* species complex in the West Pacific

**DOI:** 10.3897/zookeys.798.28375

**Published:** 2018-11-21

**Authors:** Toby S. Daly-Engel, Amber Koch, James M. Anderson, Charles F. Cotton, R. Dean rubbs

**Affiliations:** 1 Florida Institute of Technology, 150 W. University Blvd, Melbourne Florida 32901, USA Florida Institute of Technology Melbourne United States of America; 2 University of West Florida, 11000 University Parkway, Pensacola Florida 32514, USA University of West Florida Pensacola United States of America; 3 University of Hawaii at Mānoa, 2500 Campus Rd, Honolulu, HI 96822, USA University of Hawaii at Mānoa Honolulu United States of America; 4 Florida State University Coastal and Marine Laboratory, 3618 Coastal Highway, St. Teresa, FL 32358, USA Florida State University St. Teresa United States of America

**Keywords:** Chondrichthyes, DNA barcoding, Elasmobranchii, morphology, *
Squalus hawaiiensis
*, taxonomy

## Abstract

Dogfish sharks of the genus *Squalus* are small, deep-water sharks with a slow rate of molecular evolution that has led to their designation as a series of species complexes, with low between-species diversity relative to other taxa. The largest of these complexes is named for the Shortspine spurdog (*Squalusmitsukurii* Jordan & Snyder), a medium-sized dogfish shark common to warm upper slope and seamount habitats, with a putative circumglobal distribution that has come under investigation recently due to geographic variation in morphology and genetic diversity. The Hawaiian population of *Squalusmitsukurii* was examined using both morphological and molecular analyses, putting this group in an evolutionary context with animals from the type population in Japan and closely-related congeners. External morphology differs significantly between the Hawaiian and Japanese *S.mitsukurii*, especially in dorsal fin size and relative interdorsal length, and molecular analysis of 1,311 base pairs of the mitochondrial genes ND2 and COI show significant, species-level divergence on par with other taxonomic studies of this genus. The dogfish shark in Hawaii represents a new species in the genus, and the name *Squalushawaiiensis*, the Hawaiian spurdog, is designated after the type location.

## Introduction

Deep-water sharks like the dogfish sharks (Squaliformes, Squalidae) and the gulper sharks (Squaliformes, Centrophoridae) have proven confounding groups for systematists to resolve due to their highly conserved morphology, wide ranges, and patchy, infrequently-sampled distributions (Veríssimo et al. 2014; Cotton and Grubbs 2015; Veríssimo et al. 2017; Daly-Engel et al. submitted). Recent years have shown that DNA sequencing in conjunction with morphological analyses is an effective approach for elucidating the alpha taxonomy of deep-water sharks (Avise 2004; Ward et al. 2005; Last et al. 2007c; Ebert et al. 2010; Veríssimo et al. 2014; Pfleger et al. 2018). Findings from several studies have shown a mix of low genetic distances between well-established morphological species, and deep genetic splits between animals identified as conspecifics (Daly-Engel et al. 2010; Veríssimo et al. 2017; Daly-Engel et al. 2018; Pfleger et al. 2018; Daly-Engel et al. submitted).

Taxonomic delineation that incorporates DNA analysis has often relied upon consistencies among within- and between-species divergences in the barcoding gene (COI), as measured by percent nucleotide sequence variation (Avise 2004; Ward et al. 2005; Naylor et al. 2012). DNA barcoding is an effective, widely-used molecular method among taxonomists because the cytochrome oxidase I gene (COI) records a low rate of mutation compared with other loci (Avise 2004; Ward et al. 2005). While a reliable metric among distantly-related groups in which mutations accumulate consistently, COI may fail to elucidate shallow divergences among taxa with low genetic diversity. Complicating identification issues is the fact that deep-water sharks, whose cold environment results in low metabolic rates relative to other elasmobranchs, may undergo an overall slower rate of molecular evolution compared with shallow coastal species (Martin et al. 1992; Martin and Palumbi 1993). As a result, a number of investigators have found the more-rapidly evolving ND2 gene to be an effective genetic marker for estimating both inter- and intraspecific variation in dogfish sharks (Veríssimo et al. 2010; Naylor et al. 2012; Veríssimo et al. 2017; Pfleger et al. 2018).

Much work among shark systematists has focused on clarifying species delineations in dogfishes of the genus *Squalus*, an abundant, speciose, globally-distributed group of morphologically-similar, small-bodied demersal sharks that primarily inhabit circumglobal shelf and slope habitats from 100 - 1000 m depth (Compagno et al. 2005; Last et al. 2007c). Within this genus, genetic and morphological examinations have revealed a series of species complexes characterized by relatively shallow evolutionary divergences among putative species (Last et al. 2007c; Gaither et al. 2016; Veríssimo et al. 2017). Such complexes are not unusual in nature, having been observed across a variety of phyla from insects (Perring 2001) and nematodes (Chilton et al. 1995) to bony fishes (Barluenga and Meyer 2004), and have been shown to harbor “cryptic” diversity not always apparent from morphology alone (Daly-Engel et al. Submitted). Taxonomic reevaluation of *Squalus* in the Indo-Pacific and elsewhere has revealed many undescribed species that were historically lumped together under a single name (Ward et al. 2007; Naylor et al. 2012; Viana et al. 2016; Pfleger et al. 2018): although the relatively shallow spiny dogfish *Squalusacanthias* Linnaeus comprises just one wide-ranging species apart from the North Pacific (Ebert et al. 2010; Veríssimo et al. 2010), we now know that both the Shortspine dogfish shark *Squalusmitsukurii* Jordan & Snyder and the shortnose dogfish shark *Squalusmegalops* Macleay comprise global species complexes (Veríssimo et al. 2010; Naylor et al. 2012; Veríssimo et al. 2017; Pfleger et al. 2018; Daly-Engel et al. Submitted).

Recent taxonomic studies on *Squalus* have focused on *Squalusmitsukurii*, a putative circumglobal species found on continental and insular shelves and upper slopes and on seamounts between 100 and 950 m depth (Compagno et al. 2005). Re-examination of local *S.mitsukurii* stocks has revealed many new species, including four from the West Pacific alone: *S.formosus* White & Iglesias (2011), *S.chloroculus* Last, White, & Motomura (Last et al. 2007b), *S.montalbani* Whitley (Last et al. 2007b), and *S.griffini* Phillips (Duffy and Last 2007). Other revisions of *S.mitsukurii* have been done in the Atlantic using either genetic tools or morphological characters (Viana et al. 2016; Veríssimo et al. 2017), though not both (but see Pfleger et al. 2018).

Along the Hawaiian Archipelago in the Central Pacific, the Shortspine spurdog (Squaluscf.mitsukurii) is the only *Squalus* species known, aggregating in large numbers on or near the bottom at a depth of 100–950 m (Wilson and Seki 1994). Observable differences between specimens of *S.mitsukurii* in Hawaii and its conspecifics from the West Pacific first came to light during a genetic study (Daly-Engel et al. 2010), and subsequent research showed that growth (*L_∞_*, *k*) and reproductive parameters (size-at-maturity) for Squaluscf.mitsukurii in Hawaii differed from published data (putatively as *S.mitsukurii*) from other regions (Cotton et al. 2011). Together with the relative geographic isolation of the Hawaiian Islands and the high levels of endemism observed there [25% of fish species in Hawaii are endemic, the most in the Indo-Pacific region (Roberts et al. 2002; Randall 2007; Briggs and Bowen 2011)] make this population a likely candidate for redescription.

We undertook a taxonomic evaluation of *Squalusmitsukurii* from the Hawaiian Islands using molecular and morphological data, couching these in the evolutionary context of closely-related, previously-recognized congeners from the West Pacific. DNA barcoding with COI can discriminate among species in the genus *Squalus* (Ward et al. 2007), but low resolution in this marker may fail to identify cryptic diversity. We therefore supplemented DNA sequences derived from the COI barcoding gene with data from ND2, a faster-evolving mitochondrial gene with the potential to distinguish evolutionary relationships with a high degree of resolution (Avise 2004; Naylor et al. 2012). In addition, morphological and meristic comparisons were made comparing S.cf.mitsukurii from Hawaii with measurements taken from the Japanese holotype, and reported by Last et al. (2007c) and in Viana et al. (2016).

## Materials and methods

### Tissue collections

Whole specimens and genetic samples of Squaluscf.mitsukurii were collected primarily during longline surveys conducted on the insular slope around the Hawaiian Island of Oahu. Survey methods are described in Daly-Engel et al. (2010) and Cotton et al. (2011). Additional specimens and samples were collected from bottom fish surveys from Maui to Lisianski Atoll, spanning nearly 2,000 km of the Hawaiian Archipelago. Because the 2010 genetic study showed extremely low diversity, we deemed 5–10 specimens adequate for taxonomic evaluation, a number on par with other revisions in this genus (Ward et al. 2007; Pfleger et al. 2018). To that end, five whole mature females and three whole mature male S.cf.mitsukurii from Oahu were retained as voucher specimens for morphometric and genetic analyses. Small (< 1cm^3^) samples of fin or muscle tissue were taken using scissors and stored in 2 mL vials containing 1.5 mL 20% dimethylsulfoxide (DMSO) saturated salt (NaCl) buffer (Seutin et al. 1991) or >70% ethanol (EtOH).

Genetic examination of 130 tissue samples from 25–30 *Squalus* dogfish species has shown that S.cf.mitsukurii from Hawaii clustered closely with *S.nasutus* Last, Marshall, & White from Australia, *S.japonicus* Ishikawa from Japan and elsewhere, and *S.mitsukurii* from Japan (Daly-Engel et al. Submitted), and well apart from other congeners in the region; hence the current analysis focuses on these species. Because it is impossible to extract undamaged DNA from the formalin-fixed *S.mitsukurii* holotype, we referenced DNA extracted from two specimens identified as *S.mitsukurii* by expert Japanese systematist Dr. Sho Tanaka (Tanaka et al. 1975; Yano and Tanaka 1984; Yano et al. 2017) collected from Suruga Bay in mainland Japan, which is approximately 100 kilometers from the type locality of Misaki. Tissue samples of *S.nasutus* (N = 2) and *S.japonicus* (N = 8) were obtained from Australia and Japan (Appendix 1).

### Genetic analysis

DNA was extracted from fin clips using a DNeasy Blood & Tissue Kit from Qiagen (Germantown, MD). Primers were obtained from Integrated DNA Technologies, Inc. (Coralville, Iowa). PCR reactions consisting of 7 μL BioMix Red from Bioline (London, UK) at the recommended concentration, 1 μL (3 μg) template DNA, and 1 μL (1.0 μM) each primer (10 μL total PCR volume). PCR amplification on a C1000 Touch Thermal Cycler (Bio-Rad; Hercules, California) consisted of an initial denaturation at 95 °C for 4 minutes followed by 36 cycles of 1 min at 95 °C, followed by 30s at 58 °C, and 30s at 72 °C with a final extension at 72 °C for 20 minutes. DNA from two mitochondrial genes were sequenced for a total of 1,131 base pairs (bp; Table 1): Cytochrome Oxidase I (COI; 602 bp), and NADH dehydrogenase 2 (ND2; 529 bp). COI primers were standard barcoding primers by Folmer et al. (1994), and NADH 2 primers that were utilized were designed by Veríssimo et al. (2010; Table 1). PCR products were cleaned with ExoFAP (Thermo Fisher Scientific, Waltham, Massachusetts) and sequenced on an Applied Biosystems 3730XL DNA Analyzer at the University of Arizona Genetics Core.

**Figure 1.**
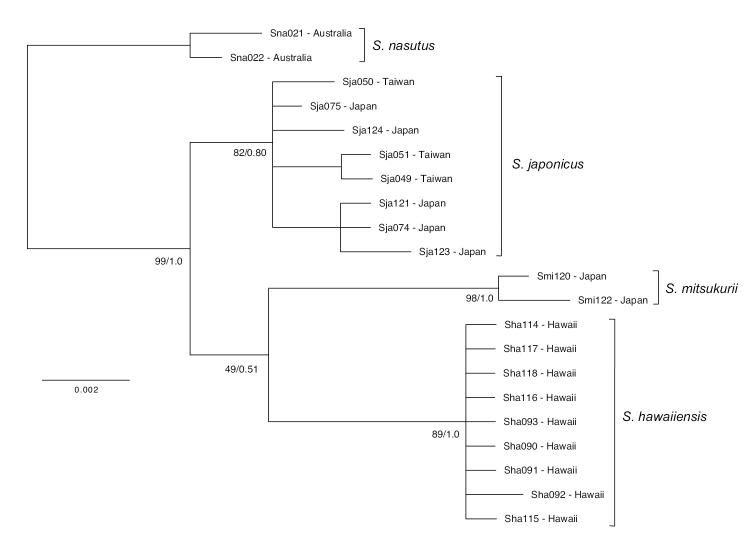
Phylogenetic tree of concatenated ND2 and COI sequences. Bayesian phylogenetic tree of concatenated mitochondrial ND2 and COI sequences for *Squalus* species used in this study, which was concordant with maximum likelihood methods. Numbers at nodes represent maximum likelihood bootstrap support/Bayesian posterior probability.

DNA sequences were trimmed in Geneious v9.1.4 (Kearse et al. 2012) and aligned using Mafft (Katoh et al. 2002) implemented in Geneious. MrBayes v3.1.2 (Huelsenbeck and Ronquist 2001; Ronquist and Huelsenbeck 2003) was used to construct Bayesian inference phylogenetic trees: first, analyses of Markov Chain Monte Carlo (MCMC) chains were run for 10,000,000 generations while sampling one tree per 100 generations. Convergence between simultaneous runs was reached when the average standard deviation of split frequencies fell below 0.01 (Ronquist et al. 2005). Following a burn-in phase of 10,000 steps (10% of generations discarded), parameter values were averaged, and posterior clade probabilities were calculated and the likelihood scores for all the topologies averaged. PhyML v3.0 (Guindon et al. 2010) implemented in Geneious was used to construct a maximum likelihood (ML) tree with 10,000 bootstrap replicates, which was compared with the Bayesian topology (Figure 1). TCS networks (Clement et al. 2000) and gene diversity statistics were generated in PopART (Leigh et al. 2014), while mutational model was calculated for each gene independently using jModeltest (Table 1; Posada 2008). Genetic distance expressed as percent sequence divergence (Table 2) was calculated in MrBayes (Huelsenbeck and Ronquist 2001).

**Table 1.** Details and diversities of genetic loci amplified in *Squalushawaiiensis* sp. n. Abbreviations: COI = Cytochrome oxidase I; ND2 = ND2 dehydrogenase 2; N = number of individual specimens included in the analysis; T_a_ = annealing temperature; π = nucleotide diversity; S = segregating sites; I = informative sites; H = number of haplotypes; h = haplotype diversity.

Gene	F primer	R primer	# bp	N	π	S	I	H	model	Citation
COI	LCO	HCO	602	20	0.0038	8	8	6	HKY	(Folmer et al. 1994)
ND2	ND2F	ND2R	529	20	0.0069	15	10	9	TRN	(Veríssimo et al. 2010)

**Table 2.** Genetic distances expressed as a percent divergence between *Squalus* species. Lower wedge is average between-species divergence in concatenated ND2 and COI genes; upper wedge is between-species divergence in each gene expressed as ND2/COI. Shaded boxes show within-species variation in concatenated sequences (top) and ND2/COI (bottom).

	* S. mitsukurii *	* S. japonicus *	*S.hawaiiensis* sp nov	* S. nasutus *
*S.mitsukurii* (N = 2)	0.09	1.07/0.30	1.01/0.50	1.70/0.70
	0.20/0.00			
*S.japonicus* (N = 8)	0.71	0.12	0.84/0.50	1.07/0.70
		0.13/0.18		
*S.hawaiiensis* sp nov (N = 8)	0.73	1.90	0.00	1.01/0.80
			0.00/0.00	
*S.nasutus* (N = 2)	1.15	0.87	0.91	0.09
				0.20/0.00

### Morphometrics and meristics

Morphological measurements were used to discriminate between Japanese *S.mitsukurii*, including the holotype as measured by Last et al. (2007c) and Viana et al. (2016), and S.cf.mitsukurii collected from Hawaii. Measurements were performed on fresh specimens in accordance with conventional techniques used for sharks (Compagno 1984), including taxon-specific adaptations (e.g. fin spine measurements) used in recent publications (Last et al. 2007c; Veríssimo et al. 2014). A suite of 82 morphological and meristic measurements were recorded for eight specimens. Measurements were taken by two readers for each individual, and the average measurement between the two readers is reported for three specimens to be designated as a holotype and two paratypes, along with the minimum and maximum values measured across the five remaining specimens. Vertebral meristic data were obtained for six specimens, including the three type specimens, using X-radiographs conducted at the Shepherd Spring Animal Hospital in Crawfordville, Florida. Dermal denticles from a male specimen (72.5 cm TL) were imaged by the Florida State University’s Biological Science Imaging Resource (BSIR) using a scanning electron microscope with Everhart-Thornley Detector (SEM ETD; FEI Nova 400 NanoSEM; BAL-TEC CPD030 Critical Point Dryer) at 15 kV, with a spot size of 3 at magnifications of 195–800×.

## Results

### Genetic analyses

**Figure 2.**
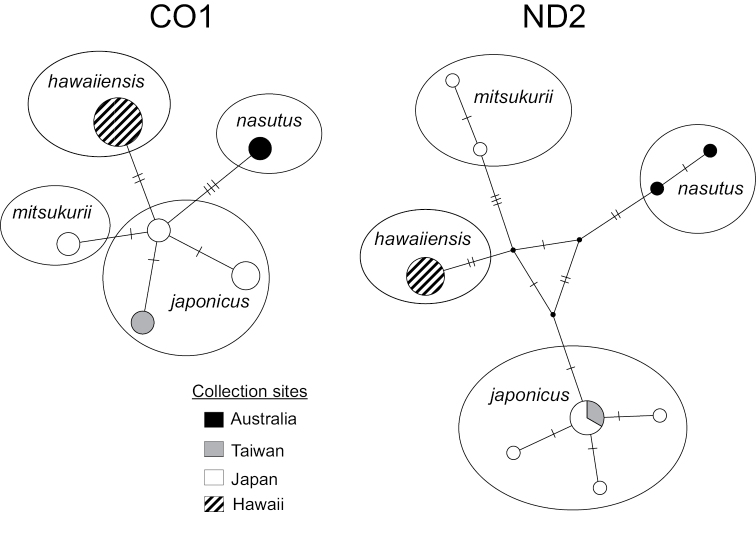
Haplotype networks for 20 individuals from four putative *Squalus* species. TCS plots show two the mitochondrial genes (COI and ND2) sequenced this study. The size of the circles or wedges represents the number of samples within each haplotype, and uninterrupted branches represent single mutational steps.

Mitochondrial DNA sampled from four conspecific shark taxa in the genus *Squalus* from the Central and West Pacific (*S.mitsukurii*, *S.nasutus*, *S.japonicus*, and S.cf.mitsukurii) clustered into four genetically distinct genetic groups with a high degree of confidence using both Maximum Likelihood (89–98% bootstrap support) and Bayesian methodology (1.00 posterior probability), except for *S.japonicus* (80% bootstrap support, 0.80 posterior probability). COI and ND2 trees were concordant, though jModeltest showed slightly different best-fit models of molecular evolution for each (Table 1), and the concatenated tree is shown (Figure 1). As expected, ND2 showed roughly twofold-higher diversity (? = 0.0069) than COI (? = 0.0038), though the evolutionary patterns they describe are similar (Table 1). TCS networks illustrate distinct genetic separation between the four taxa, with some haplotypes being closely related, but none shared (Figure 2).

Among the four species examined here, interspecific divergence across 1,131 bp of concatenated mtDNA ranged from 0.71% between *S.mitsukurii* and *S.japonicus* to 1.90% between *S.japonicus* and S.cfmitsukurii (average = 1.05±0.18%). Average pairwise genetic distance between S.cfmitsukurii and the three named species was 1.18%, greater than the average distance linking named pairs (0.91%). Intraspecific divergence ranged from 0.00% among eight S.cf.mitsukurii to 1.12% in the same number of *S.japonicus*. Such lack of diversity is consistent with a 2010 population genetic study of *Squalus* from Hawaii that recovered only eight CO1 haplotypes in 112 individuals, and only five haplotypes in the 91 sharks sampled from Oahu (Daly-Engel et al. 2010). Haplotype diversity was also low in *S.nasutus* and *S.mitsukurii*, likely because these were represented by just two samples each. Novel DNA sequences have been made publicly available via GenBank (Appendix 1).

#### Squalus hawaiiensis sp. n.

Taxon classificationAnimaliaSqualiformesSqualidae

http://zoobank.org/105A6FF0-9FFD-4425-BE9C-85019A911B25

##### Diagnosis.

**Figure 3.**
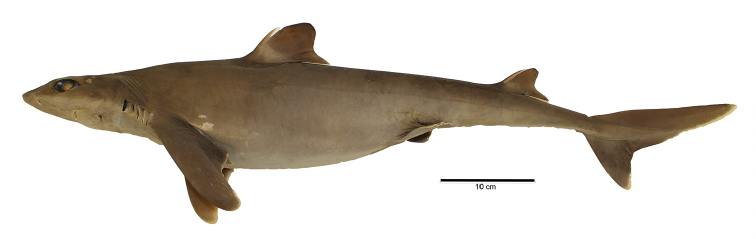
Holotype. Lateral view of *Squalushawaiiensis* sp. n. holotype (UF241161, female 750.5 mm TL).

**Figure 4.**
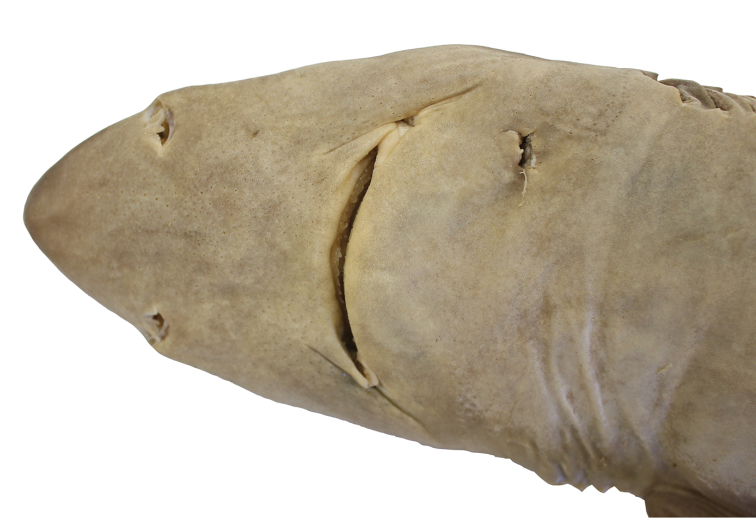
Holotype. Ventral view of *Squalushawaiiensis* sp. n. holotype (UF241161, female 750.5 mm TL).

**Figure 5.**
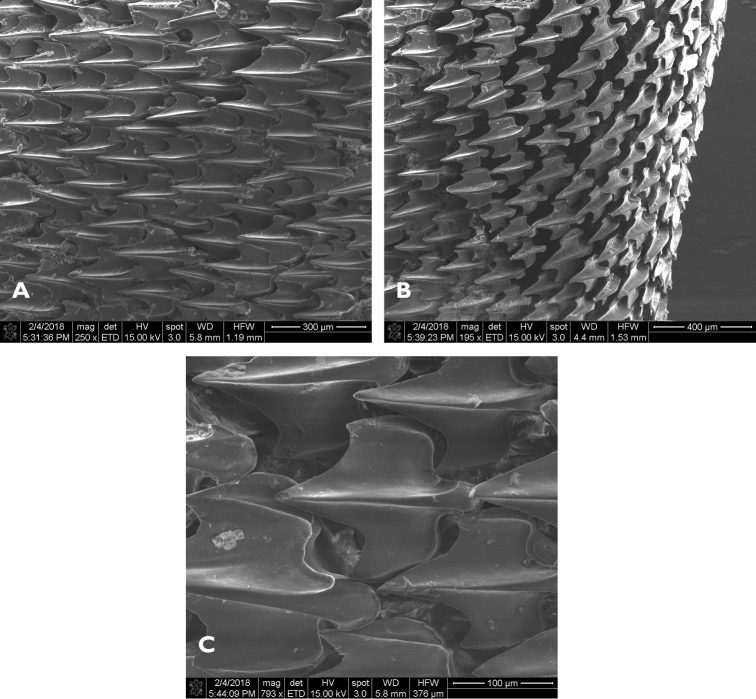
SEM images of dermal denticles. Three views of dermal denticles from adult male (TL = 72.5 cm) *Squalushawaiiensis*.

A large species of *Squalus* of the ‘mitsukurii group’ with the following combination of characters: body relatively slender, trunk height 8.7–12.4% TL (mean 10.1% TL, n=8; Figure 3); snout is angular and short to moderate in length, mouth width 1.35–1.60 (1.48) times horizontal prenarial length and pre-oral length is 1.92–2.06 (1.97) times the prenarial length (Figure 4); pre-first dorsal length 30.3–31.5 (30.2)% TL; pre-second dorsal length 63.6–67.0 (65.5)% TL; interdorsal space 26.7–30.0 (28.6)% TL; pelvic-caudal space 25.2–29.3 (27.1)% TL; relatively small, upright dorsal fins; first dorsal fin length 11.4–12.8 (12.2)% TL, height 6.5–7.8 (7.3)% TL, inner margin length 4.9–5.7 (5.4)%% TL; second dorsal fin length 10.6–11.7 (11.1)% TL, height 4.0–4.6 (4.4)% TL, inner margin length 4.3–4.9 (4.6)% TL; first dorsal fin spine length 46.6–64.6 (55.6)% of first dorsal fin height; second dorsal spine length 104.5–114.5 (109.0)% of second dorsal fin height; caudal bar triangular, extending from the caudal fork nearly to the anterior edge of the lower caudal, distinct upper caudal blotch and fringe in juveniles, upper caudal blotch diffuse in adults but extending to the posterior margin of the upper caudal fin, upper and lower caudal fins white tipped; flank denticles tricuspid (Figure 5A–C); teeth are similar in appearance in the upper and lower jaw, with numbers ranging from 26–28 in the upper jaw and 23 in the lower jaw; 41–45 monospondylous centra, 85–89 precaudal centra, 112–116 total centra; adult maximum size at least 101 cm TL.

##### Description.

**Figure 6.**
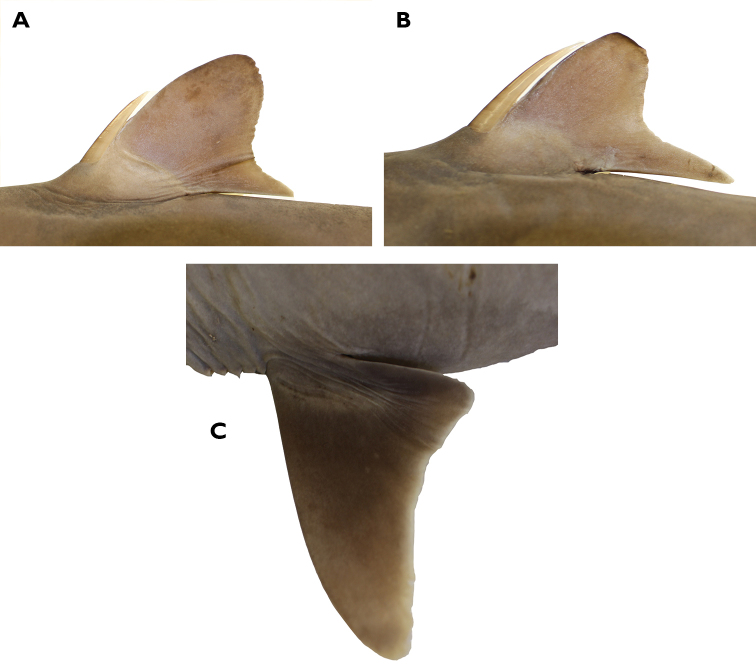
Holotype**. A** First dorsal fin **B** second dorsal fin **C** pectoral fin of *Squalushawaiiensis* holotype (UF241161, female 750.5 mm TL).

Morphometric data are provided in Table 3. *Squalushawaiiensis* sp. n. is a relatively large dogfish shark with a fusiform body, a relatively short snout, and small dorsal fins. The nape is modestly humped over the pectoral fins, particularly in large females. Head length is 21.4–23.9% TL. The snout is relatively short but angular and relatively pointed in dorsal view, with a pre-narial length that is 49–52% of the pre-oral length and 1.06–1.31 times eye length. Pre-oral length is 2.04–2.42 times the internarial space. Pre-vent length is 50.4–53.6% of the TL. Mouth width is 0.69–0.83 times the pre-oral length. Eye is large (3.9–4.9% of TL) and strongly notched posteriorly. Upper and lower labial furrows pronounced. Upper labial furrow length 1.9–2.5% TL, 24.9–33.0% of mouth width, and 19.3–24.7% of pre-oral length. Inner nostril labial furrow space is 1.89–2.27 times labial furrow length. Pre-first dorsal fin length is 30.3–31.5% of TL, pre-second dorsal space is 63.6–67.0% of TL and the interdorsal space ranges from 26.7% to 30.0% of TL. The first dorsal fin is rounded at the apex. First dorsal fin length measures 1.62–1.81 times first dorsal fin height. First dorsal fin length is 1.02–1.16 times second dorsal fin length and the height of the first dorsal fin is 1.57–1.80 times the height of the second dorsal fin. Second dorsal fin length 2.36–2.79 times the second dorsal fin height. Dorsal fin spines are stout, with the spine on the second dorsal fin typically longer (4.1–5.0%TL) than the spine on the first dorsal fin (3.6–4.6%TL). First dorsal spine length is 0.39–0.65 (mean: 0.53%) times the first dorsal fin height. Second dorsal spine length is 0.84–1.15 (mean: 1.04%) times the second dorsal fin height. The pectoral fins are well developed with an anterior margin that is 12.8–16.0% of the TL. The pectoral inner margin is 6.4–7.4% of total length and free rear tip is rounded (Figure 6A–C).

**Figure 7.**
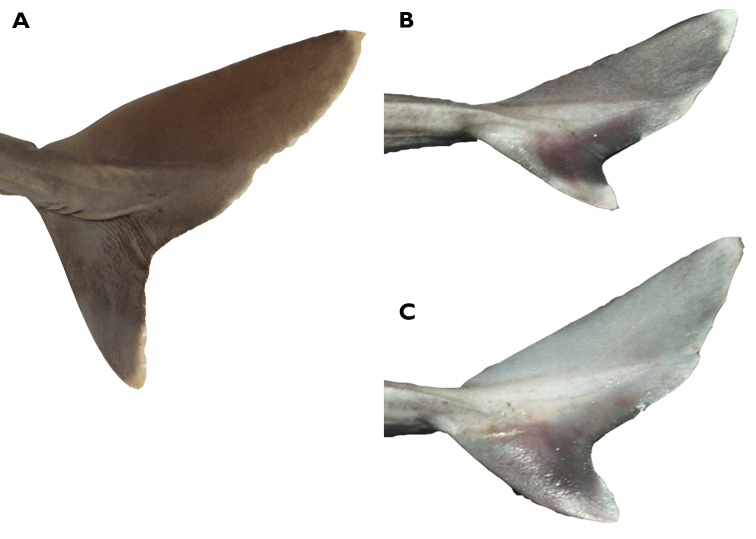
Caudal fin of *Squalushawaiiensis* sp. n**. A** Holotype (UF241161, female 750.5 mm TL) **B** fresh adult male **C** fresh adult female.

*Squalushawaiiensis* is morphologically similar to other species in the “*mitsukurii*” group. It is distinguished morphologically by a very long inter-dorsal space which ranges from 26.7% to 30.0% of TL compared to 18.7–25.5% in *Squalusmitsukurii* (Last et al. 2007a) and 23.5–24.6% in *Squalusformosus* (White and Iglésias 2011), both from Taiwan and southern Japan, and to 23.5–25.6 in *S.edmundsi*, 20.6–23.8% in *S.grahami* (White et al. 2007), 21.7–25.9% in *S.montalbani* (Last et al. 2007b), all from Australia and 22.6–26.0% in *S.griffini* (Duffy and Last 2007) from New Zealand, but overlaps with *S.chloroculus* (23.7–27.5%) from Australia (Last et al. 2007b), *S.nasutus* (24.4–28.0%) from Australia, Indonesia, and the Philippines (Last et al. 2007a) and *S.japonicus* from Japan (28.0–29.5%TL) (Chen et al. 1979). *Squalushawaiiensis* is further distinguished from *S.mitsukurii* by having smaller first and second dorsal fin lengths and anterior margins and a longer body or torso (longer pre-caudal and pre-second dorsal lengths but shorter dorsal caudal margin; Table 3). The longer torso is reflected in differences in the ranges of the following ratios between *S.mitsukurii* type specimens (reported in Last et al. 2007) and all *S.mitsukurii* measured here (N=8): pre-first dorsal length 1.45–1.73 vs. 1.01–1.16 times interdorsal space; prepectoral length 1.09–1.28 vs. 0.74–0.86 times interdorsal space; prepectoral length 1.02–1.07 vs. 0.78–0.89 times pelvic-caudal space. Based on data from Chen et al. (1979), *S.mitsukurii* has higher vertebral meristic counts (45–51 monospondylous centra, 87–93 precaudal centra, 118–127 total centra) than *S.hawaiiensis* (41–45 monospondylous centra, 85–89 precaudal centra, 112–116 total centra). *Squaluschloroculus* has a caudal bar that extends much higher on the upper caudal fin and lacks the upper caudal blotch characteristic of *S.hawaiiensis* (Figure 7A–C). *Squaluschloroculus* also has much shorter first dorsal fin spines (2.3–3.3%TL) and second dorsal fin spines (2.5–3.9%TL) than *S.hawaiiensis*. *Squalusnasutus* has a much longer snout with pre-narial lengths of 5.9–7.5%TL and pre-oral lengths of 11.1–12.7%TL compared to 4.8–5.4%TL and 9.6–10.4%TL respectively for *S.hawaiiensis*. Based on the morphometrics from Chen et al. (1979), the closely related *S.japonicus* differs from *S.hawaiiensis* in having a smaller mouth (6.4–6.9%TL compared to 7.0–8.1%TL) and shorter first and second dorsal fin lengths. First dorsal fin length in *S.japonicus* is 10.1–11.0%TL compared to 11.4–12.8%TL in *S.hawaiiensis*. Second dorsal fin length is 7.9–8.4%TL *S.japonicus* compared to 10.6–11.7%TL in *S.hawaiiensis*.

**Figure 8.**
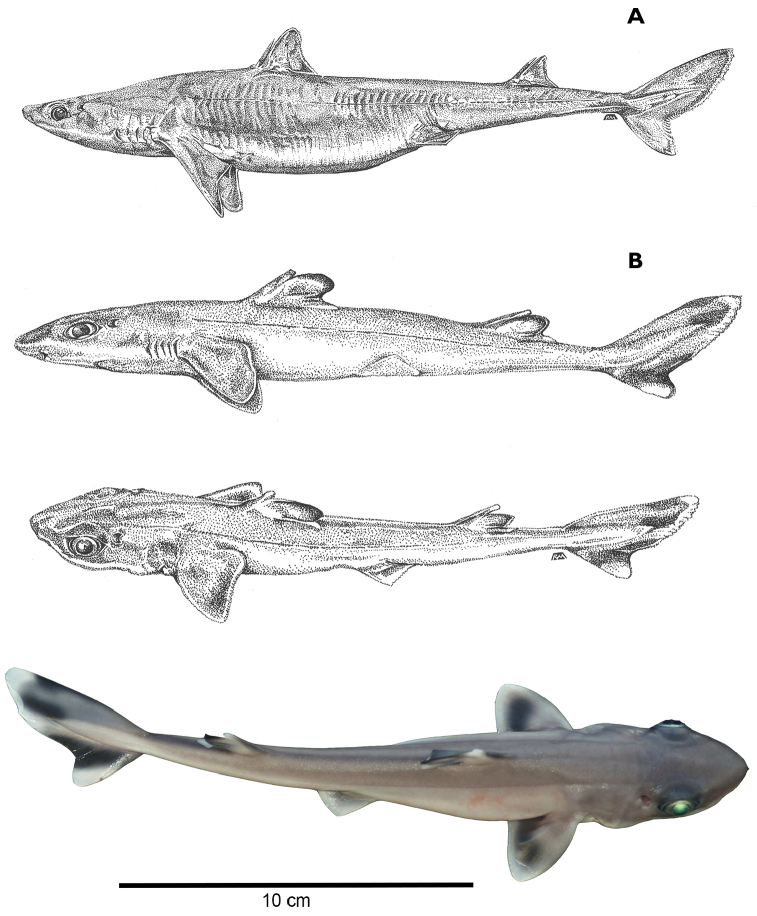
Images of the Hawaiian spurdog, *Squalushawaiiensis*. **A** Lateral view of adult female *Squalushawaiiensis*, drawing by R. McPhie **B** embryonic *Squalushawaiiensis*, lateral and dorsal views, drawings by R. McPhie **C** embryonic *Squalushawaiiensis*, dorsal view. Photo by RDG.

*Color.* In life (based on many captured specimens): dorsal surface uniformly dark gray to brown, light gray to white ventrally. Dorsal fins uniformly gray to brown with think black tips that narrow with age, free rear tips slightly paler. Caudal fin mostly dusky with a broken white trailing edge, dark caudal bar triangular, extending from the caudal fork nearly to the anterior edge of the lower caudal (Figure 8A–B). Upper caudal blotch diffuse in adults, extending to a short length of the posterior margin of the upper caudal fin, upper and lower caudal fins white tipped; pectoral and pelvic fins greyish dorsally, darker in the middle and with well-defined white posterior margin; Juveniles with much more pronounced fin markings; dorsal fins with black fringes, dark blotch in pectoral fins, caudal bar distinct on lower caudal from the fork to the anterior edge, well-defined and separated black upper caudal blotch and upper caudal fringe with upper caudal blotch not reaching the posterior margin of the upper caudal fin. In juvenile *S.mitsukurii* the upper caudal blotch is smaller and indistinct from the upper caudal fringe and the caudal bar is diagonal rather than triangular and does not reach the posterior edge of the lower caudal fin. In preservative: holotype similar, dark markings on fins faint but evident; caudal bar faint; broad, pale posterior margins on pectoral and pelvic fins well-defined. Eyes bright green in life (Figure 8C).

*Size*. Based on 197 Hawaii specimens surveyed, 156 females and 41 males (Daly-Engel et al. 2010; Cotton et al. 2011), the maximum observed length of females and males was 101 cm TL and 78 cm TL respectively. Cotton et al. (2011) reported that females reach maturity at ~64 cm TL and males reach maturity at ~47 cm TL.

*Etymology.* Derived from the type locality in the Hawaiian Archipelago

*Vernacular.* Hawaiian Spurdog

## Discussion

We found marked genetic variation across 1,311 base pairs of mitochondrial DNA separating *Squalushawaiiensis* from *Squalusmitsukurii* specimens collected from the Japanese type locality, as well as closely-related congeners from elsewhere in the Pacific (Figures 1, 2). Patterns of relatedness and inter- and intraspecific genetic distances were comparable to other phylogenetic studies on *Squalus*, including species descriptions (Ward et al. 2005; Ward et al. 2007; Naylor et al. 2012). Our data show that *S.mitsukurii* from Japan and *S.hawaiiensis* are demonstrably distinct sister species (Figures 1, 2), most closely related to *S.japonicus* from Japan and *S.nasutus* from Australia. Morphological examination also distinguished *S.hawaiiensis* from these three species by a combination of differences in the trunk length (interdorsal distance), snout length, fin and fin spine lengths, and caudal coloration. We conclude that *S.hawaiiensis* represents a novel, previously-unidentified species. A holotype and paratypes a and b have been deposited into the Florida Museum of Natural History (FLMNH catalog numbers UF241161, UF241162, and UF241163; Table 3).

**Table 3.** Morphological data from *Squalushawaiiensis* sp. n. and *S.mitsukurii* from Japan. Morphological data from type specimens of two *Squalus* species expressed as a percentage of total length (TL) in cm following the methods of Last et al. (2007b). Morphometrics from the holotype, two paratypes a and b, and the range of values from five additional specimens of *Squalushawaiiensis* sp. n. are shown; type specimens are listed with FLMNH catalog number and genetic ID from Figure 1. Morphometrics for *S.mitsukurii* were taken directly from published studies, including two independent sets for the *S.mitsukurii* holotype: ^1^Last et al. (2007b) and ^2^Viana et al. (2016). Also shown are the minimum and maximum values from Last et al. (2007b) for four *S.mitsukurii* paratypes from Japan; “Min” and “Max” represent a range of values for these paratypes, not including holotype values. Abbreviations: ♂ = male, ♀ = female, bolded numbers indicate non-overlapping size ranges between *S.mitsukurii* and *S.hawaiiensis*.

		*S.hawaiiensis* sp. n.					*S.mitsukurii* (Japan)			
		Holotype (♀ UF241161, Sha116)	Paratype^a^ (♂, UF241162, Sha114)	Paratype^b^ (♂, UF241163, Sha117)	Min	Max	Holotype^1^	Holotype^2^	Min	Max
STL	Stretched total length	774.5	628	502.5			–	–	–	–
TL	Total length	750.5	608	487.5	535	836	719	710	266	855
PCL	Precaudal length	**81.3**	**82.6**	**80.3**	**80.3**	**83.1**	76.6	77.5	78.2	79.0
FL	Fork Length	90.2	91.3	89.8	88.4	92.5	–	–	–	–
PD2	Pre-second dorsal length	**65.8**	**65.6**	**64.6**	**63.6**	**67.0**	59.8	61.0	58.6	61.2
PD1	Pre-first dorsal length	31.5	30.4	30.9	30.3	31.3	30.9	32.4	28.5	32.3
SVL	Pre-vent length	53.1	52.4	51.1	50.4	53.6	51.5	50.0	48.9	52.2
PP2	Prepelvic length	52.2	50.1	48.6	48.9	52.4	48.5	47.9	47.4	50.1
PP1	Prepectoral length	22.5	24.3	23.1	22.0	23.3	23.3	24.6	19.9	23.9
HDL	Head Length	21.8	23.9	22.9	21.4	22.4	23.4	24.2	20.9	23.5
PG1	Prebranchial length	18.3	19.2	19.1	17.9	20.7	19.5	20.4	18.0	20.1
PSP	Prespiracular length	12.2	12.9	13.2	12.0	12.9	12.8	12.8	12.1	13.3
POB	Preorbital length	7.6	7.8	7.8	7.4	7.8	7.5	7.3	7.3	7.9
PRN	Prenarial length	4.9	5.2	5.4	4.8	5.1	5.5	5.6	5.0	5.4
PINL	Pre-inner nostril	5.0	5.2	5.3	4.9	5.1	–	–	–	–
POR	Preoral length	9.9	10.1	10.4	9.6	10.2	10.8	10.3	9.4	10.6
INLF	Inner nostril-labial furrow space	4.3	4.7	4.8	4.3	4.7	4.4	4.3	4.2	4.7
MOW	Mouth width	7.7	7.6	7.2	7.0	8.1	6.2	8.6	6.3	7.5
ULA	Labial furrow length	1.9	2.5	2.1	1.9	2.3	2.4	2.5	2.1	2.5
INW	Internarial space	4.4	4.9	4.4	4.0	4.8	4.8	4.7	4.0	4.9
INO	Interorbital space	8.0	7.8	8.0	6.7	7.8	8.1	9.3	7.9	8.4
EYL	Eye length	4.3	4.9	4.5	3.9	4.7	3.4	3.6	3.8	4.7
EYH	Eye height	3.0	3.1	2.9	1.7	3.5	1.3	0.9	1.8	2.5
SPL	Spiracle length	1.2	1.4	1.7	1.2	1.6	1.2	1.3	1.2	1.5
GS1	First gill-slit height	1.7	1.6	1.4	1.5	1.9	1.9	1.7	1.6	1.7
GS5	Fifth gill-slit height	2.2	1.9	2.2	2.0	2.4	2.1	2.3	1.8	2.0
IDS	Interdorsal space	**27.8**	**28.9**	**26.7**	**28.1**	**30.0**	21.3	21.1	18.7	25.5
DCS	Dorsal-caudal space	10.2	12.1	11.4	10.9	11.6	9.8	10.6	9.9	11.2
PPS	Pectoral-pelvic space	26.2	22.9	22.8	23.6	27.7	22.5	21.8	21.3	24.5
PCA	Pelvic-caudal space	25.4	29.0	27.4	25.2	29.3	22.7	23.7	22.3	27.4
D1L	First dorsal length	12.5	11.4	11.9	11.6	12.8	14.5	13.6	12.5	15.7
D1A	First dorsal anterior margin	11.0	9.1	10.6	10.0	11.1	12.0	12.0	10.5	11.1
D1B	First dorsal base length	**7.2**	**6.2**	**6.9**	**6.4**	**7.4**	8.3	8.2	7.8	7.8
D1H	First dorsal height	7.7	6.5	7.8	6.9	7.7	8.5	9.8	4.5	8.3
D1I	First dorsal inner margin	5.5	5.2	5.4	4.9	5.7	6.3	6.2	4.9	6.4
D1P	First dorsal posterior margin	8.1	7.7	8.0	7.6	9.0	9.7	9.3	4.6	7.9
D1ES	First dorsal spine length	4.6	4.2	3.8	3.6	4.4	3.3	3.9	3.5	4.8
D1BS	First dorsal spine base width	0.9	0.8	0.9	0.7	1.0	0.8	1.0	0.6	0.8
D2L	Second dorsal length	9.4	9.7	9.7	9.2	9.9	–	–	–	–
D2L*	Second dorsal length (incl. cartilage)	**11.5**	**11.1**	**10.8**	**10.6**	**11.7**	12.7	12.3	12.0	13.9
D2A	Second dorsal anterior margin	6.9	6.5	7.3	6.7	7.4	–	–	–	–
D2A*	Second dorsal anterior margin (incl. cartilage)	**9.5**	**8.1**	**8.6**	**8.3**	**9.2**	10.2	10.2	10.4	10.7
D2B	Second dorsal base length	5.0	4.9	4.9	5.2	5.5	–	–	–	–
D2B*	Second dorsal base length (incl. cartilage)	**6.8**	**6.4**	**6.3**	**5.9**	**6.9**	7.2	7.2	8.0	9.2
D2H	Second dorsal height	4.3	4.1	4.6	4.0	4.6	4.5	6.8	3.0	4.6
D2I	Second dorsal inner margin	4.6	4.7	4.6	4.3	4.9	5.1	5.3	4.2	5.4
D2P	Second dorsal posterior margin	5.4	5.7	5.5	4.8	6.3	5.2	6.3	4.1	4.4
D2ES	Second dorsal spine length	4.1	4.7	5.0	4.1	4.6	3.8	4.2	3.8	5.0
D2BS	Second dorsal spine base width	0.8	0.8	0.9	0.7	0.9	0.7	0.9	0.7	0.9
P1A	Pectoral anterior margin	16.0	12.8	13.6	12.9	15.6	15.0	15.2	11.7	16.1
P1I	Pectoral inner margin	6.6	6.8	7.1	6.4	7.4	8.2	9.5	7.0	7.5
P1B	Pectoral base length	5.6	5.4	5.3	5.0	5.8	6.8	5.3	5.0	6.1
P1P	Pectoral posterior margin	12.2	9.9	9.9	10.1	12.3	11.0	11.7	7.6	11.4
P2L	Pelvic length	9.9	11.4	10.9	9.3	10.7	10.8	11.5	9.6	10.3
P2H	Pelvic height	3.9	3.4	3.1	3.0	5.2	5.6	–	4.0	4.9
P2I	Pelvic inner margin	4.6	5.7	5.9	3.9	6.0	5.8	6.3	2.0	3.1
CDM	Dorsal caudal margin	20.7	20.1	21.1	19.4	21.4	22.6	24.4	21.2	21.3
CPV	Preventral caudal margin	11.2	9.9	10.3	10.2	12.0	12.3	12.1	10.2	12.2
CPU	Upper postventral caudal margin	16.6	15.0	15.4	14.3	16.6	16.4	–	13.2	16.2
CPL	Lower postventral caudal margin	5.2	4.0	3.2	3.7	5.4	4.8	–	3.4	5.6
CF.W	Caudal fork width	6.7	6.7	6.6	6.5	7.2	6.7	7.0	5.9	6.7
CF.L	Caudal fork length	8.5	8.1	8.5	8.2	9.3	9.2	–	9.3	10.3
HANW	Head width at nostrils	7.2	7.7	7.6	6.5	7.3	7.7	7.3	7.6	7.7
HAMW	Head width at mouth	10.8	11.1	10.5	9.9	10.6	11.5	12.2	10.1	10.8
HDW	Head width	13.6	11.7	10.8	11.7	15.8	14.8	22.5	11.5	13.8
TRW	Trunk width	15.0	10.6	10.7	11.7	14.2	–	18.3	8.2	10.7
ABW	Abdomen width	15.1	10.9	9.6	10.0	14.4	–	15.5	6.4	9.6
TAW	Tail width	7.1	7.1	5.9	5.9	7.9	6.3	–	4.7	6.7
CPW	Caudal peduncle width	3.0	3.0	2.7	2.4	3.4	2.5	–	2.4	3.1
HDH	Head height	8.2	8.3	8.2	8.1	10.7	8.5	12.7	7.5	11.7
TRH	Trunk height	9.2	8.7	8.8	8.8	12.4	–	10.3	7.9	9.1
ABH	Abdomen height	**11.1**	**9.3**	**9.2**	**8.6**	**14.2**	–	7.7	7.7	8.4
TAH	Tail height	6.3	5.9	5.3	5.7	8.8	7.2	–	5.3	6.2
CPH	Caudal peduncle height	2.3	2.2	2.2	2.3	2.5	2.6	–	2.3	2.5
CLO	Clasper outer length		4.5	3.8	5.1	5.1	–	–	1.7	2.6
CLI	Clasper inner length		7.7	6.3	8.4	8.4	–	–	5.2	6.0
CLB	Clasper base width		1.7	1.2	1.6	1.6	–	–	0.9	1.1

The holotype of *Squalusmitsukurii* was first listed from Misaki, Japan by Jordan and Snyder (1901) before being officially described two years later by Jordan and Fowler (1903), though as in the 1901 paper, the accompanying drawing was of *Squalusacanthias*. *Squalusmitsukurii* from Hawaii was referenced by Jordan & Evermann shortly thereafter in the publication “Shore Fishes of the Hawaiian Islands” (1905), with a copy of the misattributed illustration from 1903. Little scientific investigation has been done on *Squalus* from the Central Pacific since then, with the exception of a 1994 account of the rapid depletion of the dogfish stock around Hancock Seamount in the Northwestern Hawaiian Islands as a result of bycatch in the armorhead fishery (Wilson and Seki 1994), and a more recent investigation of age, growth, and reproduction (Cotton et al. 2011). In 2010, the authors found that S.cf.mitsukurii from Hawaii has the lowest rate of both multiple paternity and genetic diversity estimated in a shark population to date, indicating that this species might have a particularly low rebound potential in the face of fishing pressure (Simpfendorfer and Kyne 2009; Daly-Engel et al. 2010).

Because taxonomic descriptions that incorporate molecular data may use different marker types, study taxa, and methods of estimating divergence, it can be difficult to directly compare genetic distances among studies, or define a genetic threshold for speciation. But a lack of shared haplotypes, plus variation between species that is generally an order of magnitude higher than variation within species, is a consistent pattern reported in many elasmobranch species descriptions (Spies et al. 2006; Ward et al. 2007; Veríssimo et al. 2014; Daly-Engel et al. 2018; Pfleger et al. 2018). Among the four closely-related species we studied, average concatenated sequence divergence between species (1.045±0.183%) was nearly fourteen times the average within-species divergence (0.075±0.026), and therefore consistent with species-level differences reported for other elasmobranchs, including *Squalus* (Ward et al. 2007; Ebert et al. 2010; Viana et al. 2016).

In addition to being taxonomically unresolved, members of genus *Squalus* are often subject to high fishing pressure as bycatch in commercial trawl fisheries, sometimes resulting in severe population depletion (Wilson and Seki 1994; Graham et al. 2001; Kyne and Simpfendorfer 2007; Dulvy et al. 2014; Pfleger et al. 2018). Furthermore, their long reproductive intervals (12–24 months) and slow growth results in a low rate of replacement (Musick 1999; Musick and Ellis 2005; Cotton et al. 2011; Cotton and Grubbs 2015), compounding the depleting effect of fishing pressure. In general, *S.mitsukurii* is classified by the International Union for Conservation of Nature (IUCN) as Data Deficient globally (Cavanagh et al. 2007), but life history parameters among *Squalus* species likely varies due to undiagnosed taxonomic variation. The combination of these variables may result in the extirpation or extinction of deep-water stocks and species before they are described by science, so taxonomic evaluation is of vital importance to ensure the survival of species that may not yet be managed as distinct evolutionary units. Further, though the barcoding gene, COI, has great utility for species identification, it may not provide sufficient resolution for diagnosing differences between organisms with low rates of molecular evolution, such as deep-water sharks. Because so many potential species remain unexamined, the name *S.mitsukurii* now represents a series of geographically distinct Evolutionary Significant Units (ESUs), each meriting its own taxonomic examination (Daly-Engel et al. Submitted).

## Conclusions

Morphological and genetic differences indicate that the dogfish shark in Hawaii represents a novel species, designated here as *Squalushawaiiensis*, the Hawaiian spurdog shark, named for the type location. Further, *Squalusmitsukurii* in Japan is subject to taxonomic confusion even among experts, and may comprise multiple distinct species, one of which likely includes the holotype. There, thorough morphological and genetic examination is warranted to elucidate the subtle differences between co-occurring populations that are morphologically indistinguishable but genetically unique. Given the number of previously-cryptic species identified in the *S.mitsukurii* complex alone, analysis of other populations will likely yield further identification of cryptic diversity within the genus *Squalus*.

## Supplementary Material

XML Treatment for Squalus hawaiiensis

## Appendix 1

**Table:** Demographic data for specimens used in this study. ID numbers refer to those used in Figure 1; GenBank Accession numbers are listed by gene (ND2/CO1); CSIRO = Commonwealth Scientific and Industrial Research Organisation Marine & Atmospheric Research (Hobart, Tasmania).

ID #	*Squalus* species	GenBank Accession #s	Source	Collector/ identifier	Origin	Latitude / Longitude	Collection date	Depth (m)	Sex	TL (mm)
Sna021	* nasutus *	MG654959.1/ MK005141	CSIRO	P. Last & W. White	West Australia, W of Shark Bay	25.0633S, 112.1483E	23 Apr 2006	340		
Sna022		MG654960.1/ MK005140	CSIRO	W. White	West Australia, W of Leander Point	29.3100S, 113.9467E	06 Feb 1991	505		
Sja049	* japonicus *	MG654922.1/ MK005129	CSIRO	W. White	Taiwan, Tashi fish market, near I-Lan (NE coast)		24 May 2005			
Sja050		MG654923.1/ MK012557	CSIRO	W. White	Taiwan, Tashi fish market, near I-Lan (NE coast)		23 May 2005			
Sja051		MG654924.1/MK005128	CSIRO	P. Last	Taiwan, Tashi fish market, near I-Lan (NE coast)		23 May 2005			
Sja074		MG654925.1/MK005130	A. Veríssimo	N. Straube	Suruga Bay, Japan					
Sja075		MG654926.1/MK005127	A. Veríssimo	N. Straube	Suruga Bay, Japan					
Sja121		MG654927.1/MG792167.1	A. Yamaguchi	Sho Tanaka	Suruga Bay, Japan		May 5 2011		F	945
Sja123		MG654928.1/ MG792169.1	A. Yamaguchi	Sho Tanaka	Suruga Bay, Japan		21 Mar 2007		F	885
Sja124		MG654929.1/ MG792170.1	A. Yamaguchi	Sho Tanaka	Suruga Bay, Japan		24 Apr 2009		M	774
Smi120	* mitsukurii *	MG654933.1/ MG792166.1	A. Yamaguchi	Sho Tanaka	Suruga Bay, Japan		21 Mar 2007		M	778
Smi122		MG654934.1/ MG792168.1	A. Yamaguchi	Sho Tanaka	Suruga Bay, Japan		10 May 2011		F	1096
Sha090	* hawaiiensis *	MG654906.1/MK005139	J.M. Anderson	J.M. Anderson	Kaneohe Bay, Oahu, Hawaii	21.4916N, 157.7525W	17 Aug 2015	360	F	58.4
Sha091		MG654907.1/MK005138	J.M. Anderson	J.M. Anderson	Kaneohe Bay, Oahu, Hawaii	21.4916N, 157.7525W	17 Aug 2015	360	F	64.8
Sha093		MG654908.1/MK005136	J.M. Anderson	J.M. Anderson	Kaneohe Bay, Oahu, Hawaii	21.4916N, 157.7525W	17 Aug 2015	360	F	63.5
Sha114		MG654909.1/MK005131	J.M. Anderson	J.M. Anderson	Kaneohe Bay, Oahu, Hawaii	21.4983N, 157.7310W	29 Jan 2016	305	M	60.8
Sha115		MG654910.1/MK005135	J.M. Anderson	J.M. Anderson	Kaneohe Bay, Oahu, Hawaii	21.4983N, 157.7310W	29 Jan 2016	305	M	57.5
Sha116		MG654911.1/MK005134	J.M. Anderson	J.M. Anderson	Kaneohe Bay, Oahu, Hawaii	21.4983N, 157.7310W	29 Jan 2016	305	F	75.1
Sha117		MG654912.1/MK005133	J.M. Anderson	J.M. Anderson	Kaneohe Bay, Oahu, Hawaii	21.4983N, 157.7310W	29 Jan 2016	305	M	48.8
Sha118		MG654913.1/MK005132	J.M. Anderson	J.M. Anderson	Kaneohe Bay, Oahu, Hawaii	21.4983N, 157.7310W	29 Jan 2016	305	M	55.5
